# The improved cortical button shows better breaking strength of sutures compared with 10 original cortical button after cyclic loading

**DOI:** 10.1186/s40634-020-00232-y

**Published:** 2020-03-28

**Authors:** Toshiaki Takahashi, Manabu Takahashi

**Affiliations:** 1 Department of Sports and Health Science, Faculty of Collaborative Regional Innovation, Ehime University, 3 Bunkyo-cho, Matsuyama, Ehime 790-8577 Japan; 2 Department of Industrial Innovation, Faculty of Collaborative Regional Innovation, Ehime University, 3 Bunkyo-cho, Matsuyama, Ehime 790-8577 Japan

**Keywords:** Breaking strength of sutures, Cortical button, Improved EndoButton, Modulus of rigidity

## Abstract

**Background:**

Suspensory cortical buttons are widely used for fixation of reconstructed ligaments during anterior cruciate ligament (ACL) reconstruction because they have high usability and a favorable fixing force. However, it is not always easy to fix a reconstructed ACL while maintaining appropriate ligament tension. Therefore, we developed an improved cortical button that provides temporary tension until suturing is completed.

**Methods:**

Button holes of our improved EndoButton are not perpendicular to the bone surface on which the button is placed, but have an angle of 45 degrees so that the button can be temporarily fixed by applying tension to the suture. The improved EndoButton and the original EndoButton (Smith & Nephew Inc., Andover, Massachusetts) were each tied to FiberWire 5/7 metric (5 M) (manufactured by Arthrex). Ten cycles of preliminary loading (0–50 N) were applied to each suture, followed by test loading (0–250 N) for 500 or 1000 cycles. Then, a tensile test was performed at a displacement velocity of 20 mm/min.

**Results:**

The breaking strength of the sutures of the improved EndoButton were tend to higher than those of the sutures of the original EndoButton after 1000 loading cycles (*p* = 0.067, d = 0.883). The moduli of rigidity of the sutures of the improved EndoButton were higher than those of the sutures of the original EndoButton after 500 loading cycles (*p* = 0.027) and remained almost the same regardless of the number of loading cycles.

**Conclusion:**

We found that compared with the original cortical button, the improved cortical button was better able to retain suture breaking strength and modulus of rigidity, regardless of the number of load cycles.

## Background

Since secure fixation of a ligament is directly associated with postoperative outcomes, various types of fixtures are used in clinical practice [1]. Various fixtures are used in anterior cruciate ligament (ACL) reconstruction, including interference screws, post screws, double spike plate (DSP) screws and double staples [2–6], and their mechanical properties have been studied [7]. The EndoButton, a suspensory cortical button, is widely used because it allows for easy and relatively strong fixation [8, 9]. If one end of a ligament has already been fixed, for example in a case where a reconstructed ACL is first fixed on the femoral side and then fixed on the tibial side, appropriate tension of the ACL needs to be maintained during ligation of the reconstructed ligament, but this is not easy with the conventional EndoButton. It is considered that the frequency of clinical use of the suspensory cortical button can be increased if more stable fixation for early loading can be secured. Therefore, we developed a new EndoButton that can fix a reconstructed ligament easily and securely by ligation while maintaining appropriate ACL tension.

The purpose of this study is to evaluate mechanical strength of this improved EndoButton compared with the original EndoButton.

## Method

**Fig. 1:**
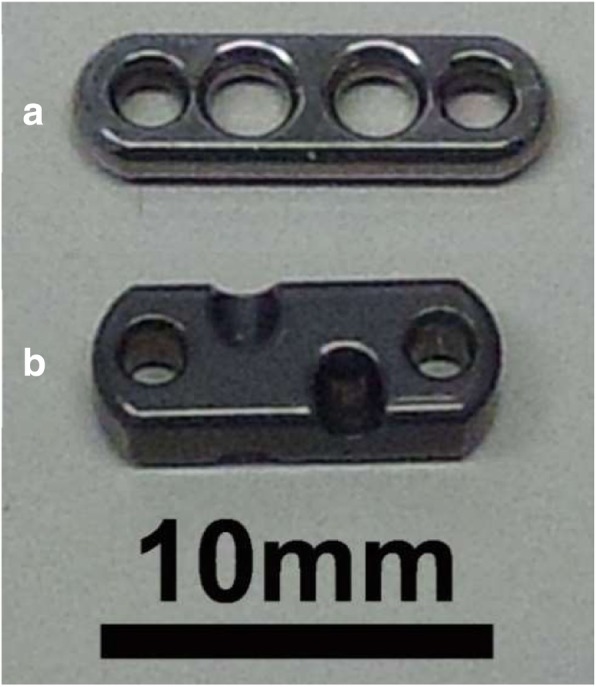
Photograph of original (**a**) and improved (**b**) EndoButton. Our improved EndoButton holes are not perpendicular to the bone surface on which the button is placed, but have an angle of 45 degrees so that the button can be temporarily fixed by applying tension to the suture. The thickness of the improved EndoButton is 2.5 mm, 1.0 mm thicker than the original one

**Fig. 2:**
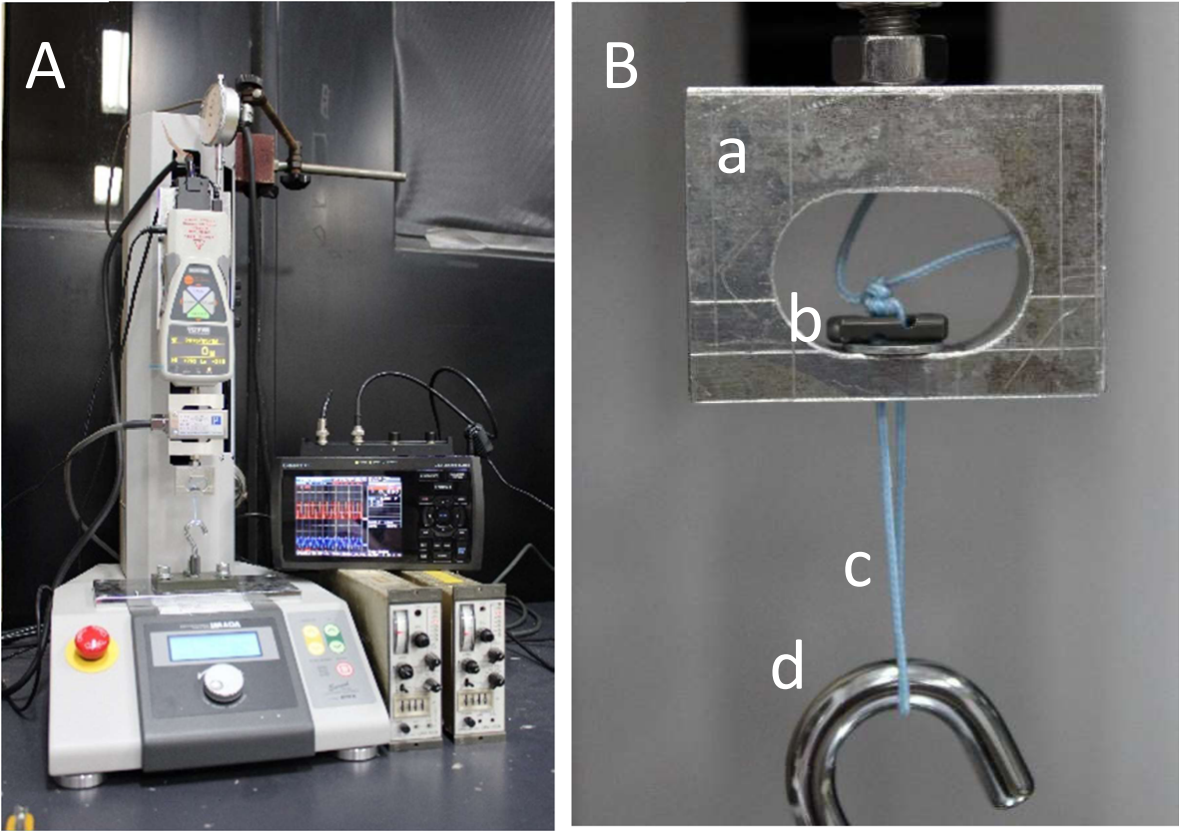
Test equipment. A Overall view (B) Testing part. a. Tension jig that simulate bone hole, b. EndoButton, c. FiberWire (5 M) d. Hook

The original EndoButton (Smith & Nephew Inc., Andover, Massachusetts) (Fig. 1a) and the improved EndoButton (Fig. 1b) and were each tied to FiberWire 5/7 metric (5 M) (manufactured by Arthrex). Button holes of our improved EndoButton are not perpendicular to the bone surface on which the button is placed, but have an angle of 45 degrees so that the button can be temporarily fixed by applying tension to the suture. Therefore, the thickness of the improved EndoButton is 2.5 mm, 1.0 mm thicker than the original one. Ten cycles of preliminary loading (0–50 N) were applied to each suture, followed by test loading (0–250 N) for 500 or 1000 cycles [10]. Then, a tensile test was performed at a displacement velocity of 20 mm/min. A force gauge (ZTA-1000 N) with a capacity of up to 1000 N was attached to the Vertical Motorized Test Stand EMX-1000 N (IMADA Co., Ltd., Tokyo, Japan) to apply displacement-controlled cyclic loading (Fig. 2). The same apparatus was used to test cyclic and tensile loading. Tensile loading was evaluated after cyclic loading. Crosshead displacement was measured with a dial displacement gauge. The minimum resolution of the dial displacement apparatus was 0.01 mm, the output setting was 2 mm/V, and the range of measurement was 20 mm. Applied load and measured displacement were converted from analogue to digital format and recorded on a personal computer. The number of specimens was 10 in each experimental condition. Cyclic load and load failure tests were performed in each examination.

A test suture was passed through the hole of the EndoButton, fixed by a double knot and then tied with four knots in the following order: square knot, granny knot, square knot and granny knot. The initial length of the suture wire was 200 mm, and the distance from the button to hook was 30 mm. Finally, the suture was cut at a distance of 10 mm from the end of the knot. The reason we used the four knots was because a suture tied with three knots was separated from the original EndoButton, but not the improved EndoButton, when a high load was applied and therefore we could not perform the test. This phenomenon also suggested that there was a significant difference between the improved and original EndoButtons in terms of the strength required to retain the suture.

The moduli of rigidity that were calculated based on data in the 100 N to 300 N range that indicated linear behavior.

### Statistical analysis

The breaking strengths and moduli of rigidity of sutures tied to the improved EndoButton were compared with those of sutures tied to the original EndoButton using the two-sample t-test, with a *p* value of < 0.05 regarded as significant (Microsoft Excel software, 2013). The effect size of Cohen’s d was calculated.

## Results

**Fig. 3:**
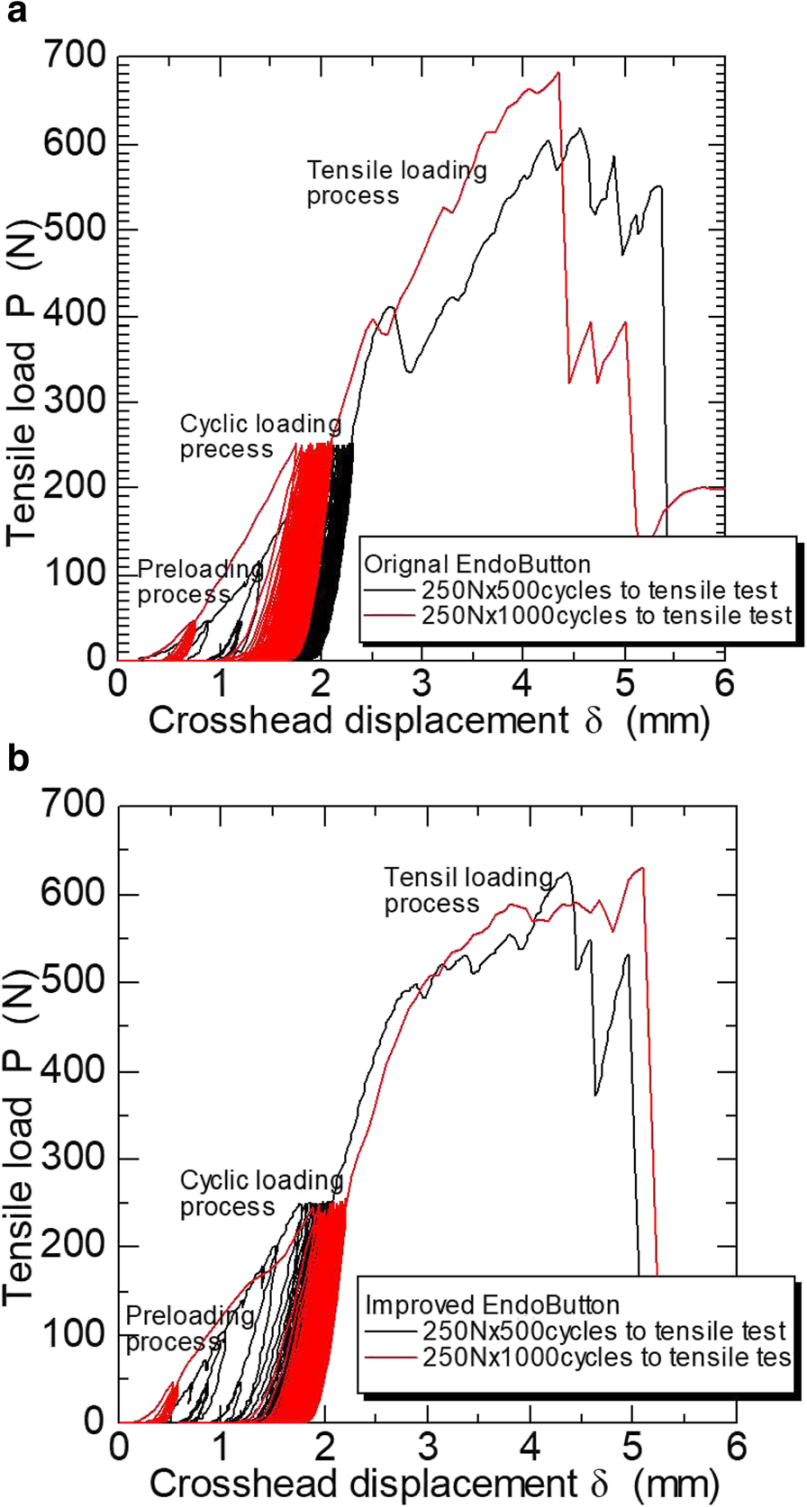
Typical load-displacement behaviors. **a** Original EndoButton (**b**) Improved EndoButton. The breaking strengths with both EndoButtons were higher than 600 N

Displacement after 500 cyclic loading (mm) under the unloaded condition was 1.96 ± 0.32 in the original EndoButton, 1.60 ± 0.22 in the improved EndoButton, as a statistical significant (*P* = 0.017). However, displacement after 1000 cyclic loading (mm) was 1.80 ± 0.40 in the original EndoButton, 1.72 ± 0.34 in the improved EndoButton (*P* = 0.678). Although the amount of displacement varied between specimens because it was affected by the tightness of the first knot, it was within approximately 1.5–2 mm after the specified cyclic loading under the unloaded condition (Fig. 3).

**Fig. 4:**
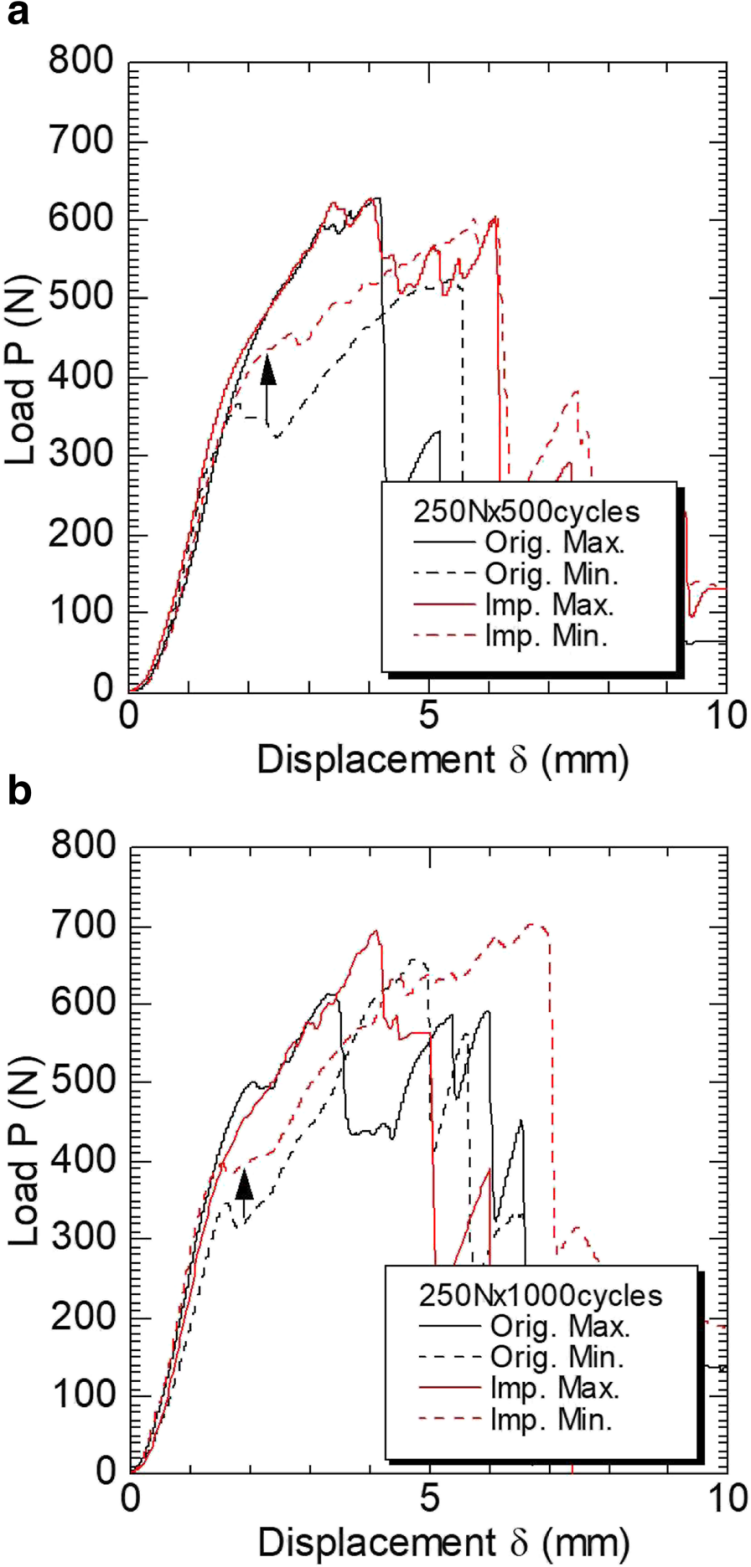
Load-displacement behaviors original and improved EndoButton after (**a**) 500 and (**b**) 1000 loading cycles (0-250N). The load-displacement behaviors of the sutures of each type of EndoButton were similar after 1000 loading cycles, as was the case with 500 loading cycles. In the case of the slip resistance of the suture is Maximum (Max.) force, both EndoButtons showed almost same resistance in the first peak. In contrast, in the case of the slip resistance of the suture is Minimum (Min.) force, the improved (Imp.) EndoButton showed higher resistance than that of an original (Orig.) EndoButton (Black arrow)

Increasing displacement resulted in decreasing load in a larger number of sutures of the original EndoButtons compared with those of the improved EndoButtons after 500 loading cycles. The sutures of the original EndoButtons also slipped at a load of around 400 N after 1000 loading cycles (Fig. 4).

While the means and standard deviations of the breaking strength (N) after 500 loading cycles were similar between the sutures of the improved and original EndoButtons, those of the sutures of the improved EndoButton were tend to higher than those of the sutures of the original EndoButton after 1000 loading cycles (Table 1). The results of the two-sample t-test that assumed equal variance gave a *p* value of 0.067 for the 1000 loading cycles, Effect size by Cohen’s d value was 0.883.

**Table 1:** Breaking strength (N) and moduli of rigidity (N/mm) of all specimens, mean and standard deviation

	Breaking strength (N)	Moduli of rigidity (N/mm)
500 cycles		
Original (*n* = 10)	646.5 ± 33.7	332.2 ± 15.6
Improved (*n* = 10)	667.5 ± 41.1	361.7 ± 25.5
*P* value	0.326	0.027
^a^d	0.559	1.396
1000 cycles		
Original (*n* = 10)	618.2 ± 51.2	341.4 ± 22.7
Improved (*n* = 10)	656.4 ± 33.5	359.1 ± 22.4
*P* value	0.067	0.142
^a^d	0.883	0.785

*P* value by two sample paired t-test

^a^d: Effect size by Cohen’s d

While the moduli of rigidity (N/mm) of the sutures of the improved EndoButton remained almost the same regardless of the number of loading cycles (Table 1). The moduli of rigidity (N/mm) of the sutures of the improved EndoButton for 500 loading cycles was in 361.7 ± 25.5, higher than that of original EndoButton in 332.2 ± 15.6. The results of the two-sample t-test that assumed equal variance gave a *p* value of 0.027 for the 500 loading cycles, indicating a significant difference at a significance level of 5%.

## Discussion

The most important finding of this study was that the improved cortical button was better able to retain suture breaking strength and modulus of rigidity, regardless of the number of load cycles, in comparison to the original cortical button. ACL fixation is associated with these post-operative complications [6, 11]. Successful fixation of the ACL depends on whether the reconstructed ACL was fixed using appropriate tension and whether the strength of the fixture was adequate.

A literature review on the outcomes of suspensory fixation and interference screw fixation reported that the side-to-side difference (evaluated using the KT-1000) was smaller in patients who received suspensory fixation, that ligament tears were more frequent in those who received interference screw fixation and that there was no difference in the clinical International Knee Documentation Committee (IKDC) scores between the two types of fixation [11]. In one study, histological evaluation of bone holes revealed that suspensory fixation provided more favorable tendon-bone healing than interference screw fixation [12].

On the other hand, Lubowitz et al. reported that there was no clinical difference between the two types of fixation when the all-inside technique was used [13]. A study in dogs reported that suspensory fixation provided better tendon-to-bone fixation than interference screw fixation [14]. A previous mechanical study revealed that strong fixation can be obtained by a combination of techniques in which the ACL is directly fixed with an interference screw and the sutures attached to the reconstructed ACL are fixed using suspensory fixation [10]. However, all of these studies reported no clinically relevant difference between the fixation types.

While there are various types of suspensory fixation, there has recently been increased clinical use of an adjustable-loop device, a type of cortical suspensory device [15–17]. In procedures using suspensory fixation, the possible causes of ACL loosening include ACL fixation with low tension and slipping of sutures off the fixture over time.

Some mechanical studies that compared adjustable-loop devices and fixed-loop devices reported that the latter showed higher breaking strength and less displacement [18–25]. On the other hand, other studies reported no difference in either parameter [26, 27]. One clinical study reported no differences between the two types of devices in evaluations using the KT-1000 [28].

The EndoButton is a basic cortical suspensory apparatus that is categorized as a fixed-loop device, and the consensus based on mechanical studies is that such devices provide favorable fixation. Adjustable-loop and fixed-loop devices have the same shape but the artificial ligament component differs in terms of spinning methods and materials.

To ensure stable post-operative outcomes, it is important to establish a fixation technique that provides a high breaking strength and modulus of rigidity and reduces displacement. Hopefully, such a technique will enable all surgeons to fix a reconstructed ACL with the same degree of tension regardless of their skill and experience.

Therefore, considering that it is important to allow for ligation with tension applied to the suture in order to ensure tension of the reconstructed ACL, we developed a modified EndoButton whose structure prevents sutures from slipping on the button when a large load is applied on the ACL after surgery (Fig. 2b). The improved EndoButton has a specific feature that allows temporary fixation of the reconstructed ACL by simply applying a transverse force to sutures after they are passed through the button holes (Fig. 1b). Since the button holes are obliquely angled in relation to the button surface, temporary fixation can be obtained by contact between the suture and button. Since the button facilitates suturing while tension is maintained, it can reduce postoperative loosening of the ACL.

While the moduli of rigidity of the sutures of the improved EndoButton were almost the same regardless of the number of loading cycles, those of the sutures of the original EndoButton increased with higher numbers of load cycles. The test results show that the original EndoButton most likely causes slipping and loosening of sutures in the early postoperative period which represents after 500 loading cycles, resulting in loosening of the ACL, but the improved EndoButton demonstrates increased ability to retain sutures in the early postoperative period.

It has been reported that adjustable-loop devices have the advantage of providing all-inside fixation, but their mechanical strength is slightly inferior to that of fixed-loop devices [19, 25, 26]. We plan to evaluate and compare the mechanical strength of our improved Endobutton in combination with an adjustable-loop device and fixed devices.

In clinical settings, we should consider using a quantitative tension device for temporary fixation of reconstructed ACLs, and perform ligation after removing the tension device.

Since the improved EndoButton was associated with more favorable breaking strength and modulus of rigidity than the original one, we consider that it can be used clinically not only for ACL fixation on the tibial side during ACL reconstruction but also for fixation or ligation of other ligaments under tension during reconstruction procedures.

Regarding the strength of this study, we developed a new EndoButton that can fix a reconstructed ligament easily and securely by ligation while maintaining the appropriate ACL tension. The improved EndoButton was superior to the original EndoButton in terms of both the breaking strength of the sutures after 1000 loading cycles, and the modulus of rigidity of the sutures after 500 loading cycles.

### Limitation

This study could not directly predict clinical outcomes because it used a simplified model consisting of a suture and a fixation device, and did not use any biological bones or ligaments for mechanical evaluation. Future mechanical studies need to be conducted using femurs, tibiae and tendons of the lower extremities of animals such as swine to simulate ACL reconstruction procedures.

The potential clinical relevance of the present study is that the improved cortical button is a beneficial and easy-to-use ligament fixture for ACL reconstruction.

## Conclusion

In this study, the improved EndoButton retained breaking strength and modulus of rigidity regardless of the number of load cycles, and was significantly superior to the original EndoButton in terms of breaking strength and modulus of rigidity.
